# Learning and coping through reflection: exploring patient death experiences of medical students

**DOI:** 10.1186/s12909-019-1871-9

**Published:** 2019-12-04

**Authors:** Travuth Trivate, Ashley A Dennis, Sarah Sholl, Tracey Wilkinson

**Affiliations:** 1 Bangkok Metropolitan Administration Hospital, 514 Luang Road, Pomprabsattrupai District, Bangkok, 10100 Thailand; 2 Office of Medical Education, Billings Clinic, 801 N. 29th St. Billings, Montana, 59101 USA; 3 Business School, Edinburgh Napier University, 219 Colinton Road, Edinburgh, EH14 1DJ UK; 4 Centre for Anatomy and Human Identification, University of Dundee, Dow Street, Dundee, DD1 5EH UK

**Keywords:** Patient death, Reflection, Coping strategies, Medical students, Student support

## Abstract

**Background:**

Existing studies have explored many aspects of medical students’ experiences of patient death and propose the importance of faculty support for coping. However, UK-based literature on this subject and research concerning learning through reflection as part of coping are relatively limited. This study, through the lens of reflection, aims to explore students' experiences with patient death in a UK context. These include coping strategies, support from faculty following patient death and the relationship between these experiences and learning. Our research questions were:

- How do medical students cope with and learn from their experiences?
- How does support from ward staff and the medical school help them cope with and learn from these experiences?
- How can students best be supported following patient death?

**Methods:**

We employed narrative inquiry to explore how medical students made sense of their experiences of patient death. Twelve students participated in our study via an online narrative questionnaire. Thematic analysis and complementary narrative analysis of an exemplar were applied to address our research aim.

**Results:**

Coping strategies comprised internal and external strategies. Internal strategies included (1) re-interpretation of the death into a meaningful experience including lessons learned; (2) normalization; (3) staying busy and (4) enduring negative emotions. External strategies included speaking to someone, which was found to influence normalization, and lessons learned. Both satisfactory and unsatisfactory support from ward staff was identified. Satisfactory support was characterized by the inclusion of emotional and professional support. Unsatisfactory support was often characterized by a lack of emotional support. Narrative analysis further demonstrated how the experience with patient death was re-interpreted meaningfully. Students suggested that support should be structured, active, sensitive, and include peers and near-peers.

**Conclusion:**

Many coping strategies, internal and external, were employed in students’ experiences with patient death. Student reflections, enhanced by support from ward staff, were shown to be important for learning from patient death. We encourage faculty to have regular sessions in which medical students can reflect on the death incident and discuss appropriately with others, including peers and near-peers.

## Background

During clinical placements, medical education literature suggests that the death of a patient can be an emotionally significant event for medical students [1–9]. These emotions can be negative, such as fear [1], guilt [1–4], sadness [2–8], shock [2, 7–9] and anger [3, 8], or be positive, such as feeling uplifted [2, 6]. First experiences with patient death have been reported to be the most memorable [7], moving, painful, and persistent in thoughts and memories [6]. The transition period from preclinical to clinical years is a factor that heightens the strong emotions evoked by patient death. This transition period may increase the difficulty for medical students in identifying their roles [5] and responsibilities [9] when encountering their first experiences of patient death. Learning to cope or to regulate emotions under stress [10] when faced with patient death is a key skill that medical students must learn [11–13]. Therefore, although potentially difficult, these early experiences also provide significant opportunities for learning.

### Reflection: a way to learn from experiences in clinical practices

One way to explore these learning experiences is through the lens of experiential learning theory and reflection. Experiential learning theory supports clinical-based learning insofar as it suggests knowledge can be shaped through experiences [14]. In the experiential learning cycle as defined by Kolb, reflection is a fundamental component [15, 16]. Atkins et al. [17] proposed that reflection involves first, the identification of uncomfortable feelings and thoughts; second, a critical analysis of those feelings and thoughts; and third, the development of a new perspective on learning [17]. Therefore, reflection may be a helpful mechanism to explore not only how individuals learn to cope, but also how they learn more generally from difficult clinical encounters such as patient death. According to General Medical Council outcomes for graduates 2018 [18], reflection is regarded as a coping strategy necessary for physicians to recover from difficulties in clinical practice.

### Coping strategies when experiencing patient death

Various coping strategies adopted by medical students have been identified in the literature (see Table 1). However, there is limited medical education research that explores coping through the lens of learning. One example is Kelly et al. [2], who explore both the coping strategies and the learning experiences of medical students as they reflect on their early experiences of patient death. For example, they discuss both students’ professional emotional responses and academic knowledge that emerge through reflection.

**Table 1:** Studies providing data about medical students’ coping strategies following patient death

Authors (year)	Country	Number of participants	Study method	Coping strategies identified
Firth-Cozen et al. [19]	UK	264	Questionnaire	- Rationalized/ accepted (23.6%) - Carried on with patient care (22.5%) - Talked to others (21.7%) - Dismissed the episode (13.3%)
Ratanawongsa et al. [5]	USA	32	Interview	Verbal: discussing the experiences with family, non-medical and medical friends. Non-verbal: exercise, writing, music therapy, and prayer.
Rhodes-Kropf et al. [7]	Canada	38	Quantitative questionnaire and interview	Verbal: Talked to others (76%), talked to other students (44%) and talked to their [own] significant others (27%) Non-verbal: movies and reading (12%), focused on work and study (12%) and prayed (12%).
Kelly et al. [2]	Canada	29	Interview, focus group, or written questionnaire in a narrative format	- Contemplated their life value - Rationalized - Turned the event into a learning experience. For example, learned how the pulmonary embolus was diagnosed.
Jones et al. [8]	UK	131	Qualitative questionnaire	On UK clinical attachments - Talked to family and friends (50%) - Talked to doctors (26%) - Talked to nurses (8%) On electives - Talked to family and friends > local people they were working with
Pessagno et al. [3]	USA	20	Interview	- Talked to others: family and friends and senior medical team members such as residents - Carried on with work - Accepted and dealt with negative emotions - Participated in rituals after death - Cried - Turned to religion
Smith-Han et al. [9]	New Zealand	53	Interview	- Discussed cases with colleagues and friends - Turned to something else such as exercise, a hobby or a drink

### Support from medical team and faculty

One main feature that can also be drawn from Table 1 is that a common coping strategy is speaking to someone (‘support seeking’ in the broader literature [10]). Talking to somebody, especially attending physicians or consultants, provides students with a chance to discuss and debrief [2, 8]. A discussion after patient death does not always need to be initiated by the student to be of value for coping. Some studies have reported that a discussion that was actively initiated by the staff member was especially helpful for students [3, 9]. Discussion has been found to lessen students’ emotional distress (e.g. fear, anxiety, guilt) [20] and bring a sense of closure [5]. Importantly, from an experiential learning perspective, the literature suggests that a discussion about patient death helps students reflect and learn from an academic perspective, from an emotional regulation perspective, and in the development of a professional identity [2, 5]. For example, Ratanawongsa et al. [5] found that learning how other more experienced team members reacted to a patient death enabled medical students to compare their own responses and assess whether they were appropriate. Other literature suggests that, in terms of the development of professional identity, a discussion following patient death has supported students to “engage in the difficult tension between emotion and professionalism” ([2], p.426). In other words, team interaction enhances students’ reflexivity as an interactive and ‘feed-forward’ orientation process to support the development of mature and professional responses.

To enhance the richness of the literature, our study explores students’ experiences with patient death through the lens of reflection. These experiences include coping strategies, support from faculty following patient death and the relationship between these experiences and learning. Furthermore, we provide recommendations for the systems supporting students following patient death. Our research questions were:

Through reflecting on their experiences with patient death during clinical placements:

- How do medical students cope with and learn from their experiences?
- How does support from ward staff and the medical school help them cope with and learn from these experiences?
- How can students be best supported following patient death?

## Methods

### Theoretical perspective

This qualitative study is underpinned by a social constructionist epistemology. Social constructionist epistemology means that knowledge is subjective in nature, and that multiple realities exist based on each individual’s way of knowing, or knowledge construction through his/her interactions with people [21]. Social constructionism is characterized as an interpretivist paradigm, which is a research paradigm that seeks to understand phenomena through gathering and interpreting people’s experiences [21]. A particular phenomenon can be experienced differently by different people based on their social-relational contexts; therefore, the interpretations can be varied [22]. For example, realities, or experiences with patient death in this regard, can be many according to different interactions between students and patients, relatives, ward staff, colleagues, etc.

### Methodology: narrative inquiry

Narrative inquiry is a qualitative methodology that looks for an understanding of a particular experience through analyzing personal narratives or stories [23]. It is also seen as the first and foremost methodology for learning about personal experiences [24]. Narrative inquiry arguably aligns with an interpretivist research paradigm and with social constructionism, because it deals with an interpretation of life events, both from the narrator and the researcher perspective [25]. The essence of a narrative account is not about a truthful reproduction of the past world, but about an interpretation and a construction of the narrator’s world that connects his/her past, present, and imagined future [26].

### Method: narrative questionnaire

Narrative data can be generated in many ways: interviews, naturalistic communication, field notes and narrative writing [27]. A narrative questionnaire is a form of narrative writing that, when carefully designed, can yield rich information full of various aspects of an experience through storytelling (e.g. the setting of the situation, emotion, memory and action) [22]. In their research study, Rees et al. [22] utilized a narrative questionnaire to ask medical students about professional dilemmas that they had experienced during their clinical years. Their questionnaire included items around the dilemma the participants had experienced. For example, they asked about a brief essence of the dilemma, the location, the people involved, the actions done and the feelings about the experience. Each question was followed by a free-text box for the participants to write their answers. We used their format to inform the design of our narrative questionnaire.

#### Questionnaire design

Adapting the questionnaire wording from previous studies concerning difficult experiences in clinical practice [7, 22], the questions were centered around the “most memorable” experience following patient death. Prompts were provided with the questions to promote reflection and encourage self-awareness in the narratives [28]. Because we aimed to understand the experience with patient death through student reflection, our prompts included what the participants had learned from the experience. Participants also completed a few demographic questions to enable characterization of our participant sample. Please see Additional file 1 for the question items in our questionnaire.

#### Study procedure

After ethical approval was received from the University of Dundee, the study was conducted over the summer of 2016 (June–September 2016).

#### Sampling and recruitment

We used a convenience sample for this study where subjects were selected because of their convenient accessibility and proximity. Our subjects were 4th and 5th year medical students at the University of Dundee. In order to participate, they needed to have had at least one experience with patient death on their clinical placements. The definition for ‘experience of patient death’ was quite broad. For example, this could have been an experience where students were interacting with a dying patient or had interacted with a patient to find out later that they had passed away. The students were invited to participate through multiple recruitment strategies, including: (1) email; (2) e-notices on virtual learning environments; (3) physical notices on notice-boards; (4) snowballing (existing participants recommend or help recruit future participants) and (5) medical school social networking (e.g. Medblogs). Through the invitation, which provided information about the study, students could follow a link to the online questionnaire. The narrative questionnaire (with the participant information sheet attached) was created and disseminated within the online survey software Bristol Online Survey (BOS). The University of Dundee had a subscription to BOS enabling researchers free access to the easy-to-use platform where researchers can produce, distribute and analyze surveys. Participants were informed that by completing the questionnaire they were consenting to participate, and that confidentiality and anonymity would be maintained in any data reports. They were also provided with supportive resources and the researchers’ contact information on the debriefing page of the questionnaire.

### Data analysis

#### Thematic analysis

Thematic analysis is a systematic approach to a set of data to identify key issues, patterns or themes and the association among them [29, 30]. This analysis allowed researchers to identify, sift and sort key issues in the data according to the research questions, or pre-existing interests [29, 30]. Thematic mapping, which is a process in thematic analysis, also supported the visualization of links and associations between themes [29, 30]. We used thematic analysis to explore reflections on coping strategies, the support students received, and the association between these issues and learning experience.

Initially, a sample of responses was used to identify themes and subthemes through discussion (TT and AD). Using these themes and subthemes, TT then developed an initial framework. The full team (TT, AD, TW, and SS) then met to discuss the data and the first draft of the coding framework. The framework was further iteratively revised to achieve the final framework, which was used to code the entire data set. After coding, TT explored patterns and connections between themes and subthemes to support the final interpretation of the data.

#### Narrative analysis

We chose to use Labov’s framework for narrative analysis because it is a widely recognized approach that equally emphasizes both the narrative content and the way the narrative is told [26]. In Labov’s framework [31], the narrative is organized into parts, including: (1) Abstract (the indication that there is something to tell); (2) Orientation (the description of time, place and person); (3) Evaluation (the narrator’s indication on why this is worth telling and his/her communication of emotions); (4) Complicating action (a sequence of actions representing the core of the story); (5) Resolution (the final event in the story) and (6) Coda (the indication that the story is finished and the effect of the story on the narrator). By this means, we put a particular focus on steps 3–6 in order to understand students’ reflections on their experiences and what they learned.

Additionally, narrative analysis can be further conducted by identifying emotional words and other dramatic devices such as negatives, intensifiers and repetitions to capture affective elements in the incident the student reflects upon [22]. Therefore, we applied both narrative analysis approaches informed by Labov [31] and Rees et al. [22] on one exemplar to complement the results derived from the thematic analysis.

## Results

### Participants

Twelve medical students completed the online questionnaire, with narrative responses averaging around 400 words per narrative. Participants’ characteristics are shown in Table 2**.**

**Table 2:** Characteristics of 12 medical students who participated in the online questionnaire

Characteristic	Number (%)
Gender	
Male	8 (66.7)
Female	4 (33.3)
English as first language	8 (66.7)
Year	
Year 4	8 (66.7)
Year 5	4 (33.3)
Age in range	
20–24	9 (75)
25–29	2 (16.7)
30–34	1 (8.3)

### Themes within students’ narratives about patient death experience

The findings from thematic analysis identified four major themes within students’ experiences of patient death that related to the overarching research questions. These themes included: (1) most memorable experience of patient death; (2) the aftermath of patient death; (3) perceptions of support following patient death and (4) suggestions for support. The findings from these themes will be discussed in the context of each research question.

### Coping and learning from experiences with patient death

The deaths described by participants as the most memorable included their first patient death, unexpected deaths, and the expected death of a patient with whom the student had a personal connection. When confronted with the patient death, participants identified their affective responses towards both the patient and the other individuals involved, such as co-workers and family members of the patient. There were a range of affective responses towards patients. Some of these feelings were negative (e.g. sadness, guilt), some were a mix of both positive and negative, while others were ambiguous and could be interpreted as either positive or negative. In relation to the patients’ families, participants often reported feeling empathetic towards the family members. In response to other members of the healthcare team, participants reported trying to identify whether their own feelings were in alignment with their colleagues (see Table 3).

**Table 3:** Quotations related to affective responses to the patient and other individuals

Affective responses towards the patient	Quotations
Negative feelings	“I regretted I did not have the power to terminate such a painful existence she had been experiencing” (student no. 1). “It was like a heart-breaking scene in the theatre” (student no. 2).
Mixed positive and negative feelings	“When she died there was a sense of relief that her suffering was over. However, the sense of grief and guilt did come [when we realized] that we couldn’t do anything to save her from septic shock” (student no. 3).
Ambiguous feelings	“In terms of how I felt, I was surprised at how rapidly the patient deteriorated (going from being fairly well with a planned discharge to collapse and death within minutes) [...] Other than that I’m fairly sanguine about the death, as I don’t know clinically how it could have been foreseen or avoided” (student no. 4).
Affective responses to other individuals	Quotations
Empathizing with patient family	“All they knew was that the patient had collapsed and not that he was very, very ill. This meant that when they arrived they were completely taken by surprise by the news that he had died, and when they walked onto the ward they were […] quickly ushered into another room, which must have been confusing and upsetting for them” (student no. 4).
Feeling the healthcare team might/might not be experiencing the same emotion	“I saw tears in a Year 5’s eyes” (student no. 5). “Everyone in medicine is so hardened by deaths and it came like nature to them, but not me who had that first experience” (student no. 6).

Participants also reported what happened after these experiences. Some participants reported being affected by the experience for a period after the event transpired. For example, two participants reported experiencing recurring dreams:

*“One of the ways it affected me was he was in my dreams for several days after that” (student no. 5).*

Participants also discussed some of the coping strategies they used in response to the experience. These strategies could be categorized as external and internal coping strategies. We defined internal coping strategies as internal processes that students individually engaged in to achieve their own resolution of the experience. On the other hand, we defined external coping strategies as those where students shared the experience externally with others, for example, through an informal or formal discussion.

Participants identified several internal coping strategies. For example, some participants discussed turning to religion, interpreting the death in the light of religion to help resolve their experiences with patient death. One participant said:

*“I coped with this by interpreting it in a Christian way (as I remembered it in my youth). In this way all I had experienced took on a theological meaning rather than one of a directly existential and evidential decay with no after life” (student no. 7).*

Some students employed a personal process to turn the experience into a lesson learned for personal life. For example, one of our participants perceived the patient death as a reminder to live life to the full:

*“Life is indeed a short episode and we should try to live to the full and be thankful to people who supported us” (student no. 1).*

The lessons learned were not limited to personal life; participants also reflected on their experience through the lens of their professional role. One participant reflected on the experience after seeing a patient die suddenly on the floor of the ward:

*“I do however feel that the situation could have been managed much better as there were several areas that should have been dealt with more effectively. [ … ] In retrospect, although there was clearly human tragedy involved. I believe being exposed to this has made me a better medical student, and I feel fortunate for having been in the "right" place at the right time” (student no. 4).*

Other strategies for coping were also identified. For instance, some students shifted their attention from negative emotions after the death by being busy with other tasks:

*“I coped by staying busy with other responsibilities. Staying busy made it easier to accept his death” (student no. 7).*

Some students reported just enduring the emotion without sharing the experiences with anybody. One participant said,

*“I just handled my sadness internally without discussing his death with anyone. [...] I was always quiet about my thoughts and the recurring dreams [about the patient who died]. I actually always enjoyed waking from them and being so aware of having had the experience” (student no. 5).*

We also found students employed normalization as an internal coping strategy. This was shown when they recognized that their emotional responses towards patient deaths were normal. Regarding external coping strategies, it is interesting that the majority of participants mentioned speaking to someone at work or in the family about patient death. Of note, many spoke to peers and/or near-peers.

**Fig. 1:**
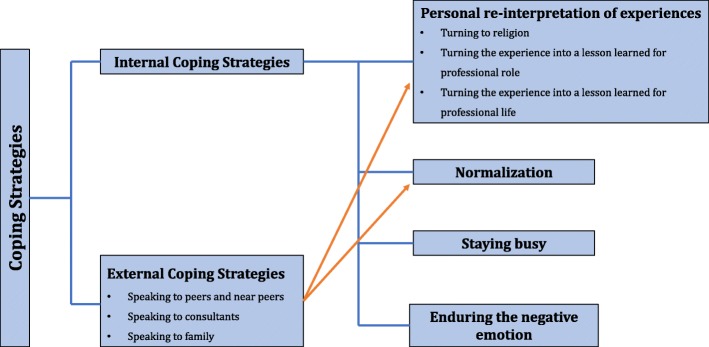
Themes related to students’ coping strategies for patient death experiences

Through the analysis, we found several instances where external and internal coping strategies co-occurred, suggesting a relationship between these themes. In particular, we found that an interaction with colleagues influenced the students’ coping mechanisms following patient death (see Fig. 1) in relation to normalization and reinterpretation of the death experience as a lesson learned.

When participants spoke with someone, they often reported that this helped normalize their feelings about the experience. One participant wrote in relation to speaking to a colleague:

*“[It] probably made it [a] more positive experience as [I] recognized [I] wasn't alone in my feelings and that this was [a] normal response to the scenario” (student no. 9).*

In addition to normalization, we found that speaking to someone on the ward promoted the re-interpretation of negative emotions into learning opportunities. This can be seen in the reflection of a student who was upset about an advanced-stage cancer patient and so left the ward when a consultant came to break the bad news to the patient. A junior doctor then spoke with them about their behavior, suggesting that in future they should not avoid the experience because there was a lot they could learn:

*“He asked me why I [had] vanished while the consultant [ … ] came. He suggested vanishing wouldn’t help me with anything, and “don’t be chicken when it comes to ward rounds”. I felt his comment was [very] strong, not in terms of humiliation, but in terms of pointing out [a fact] about myself. If I hadn’t vanished during the ward round, I would have seen how the bad news was brought to light. I did miss an experience I shouldn’t have missed [...] The foundation doctor I spoke to was a great role model” (student no. 2).*

The student no. 2 suggested that the foundation doctor knew it was unpleasant to witness the patient being told bad news. However, he suggested that the student embrace their negative emotions and be present during the ward round, which the student found helpful advice. Furthermore, the student’s comments highlight the interplay of both personal and professional aspects of patient death experiences that students must learn to negotiate. In the comment below, we see how support from colleagues helped students negotiate the personal and professional aspects of patient death:

*“I coped by talking to a ward sister who seemed to know how I felt. We didn't spend too long chatting because we knew we had to move on [with doing our jobs]. It helped me separate the personal feelings from my work. I believe everybody is affected by patient death in some way, but [that] one of our patients died doesn't necessarily mean we can't save the others” (student no. 3).*

### Impact of support from ward staff and medical school on coping and learning

There were two themes for students’ perceptions of support following patient death: (1) Support received from ward staff and (2) Support received from the medical school. The former focused on participants’ responses towards support they received from people with whom they worked on the ward, which included consultants, registrars, foundation doctors, colleagues, nurses and other medical staff. The latter concerned the responses towards support from academic staff who were not involved in the ward setting where the experience took place.

In terms of ward staff, there was a range in students’ perception of how supportive the ward staff were; some people felt that it was satisfactory, while others did not. In instances where the support was satisfactory, personal emotional support from peers and near-peers was a common feature. For instance, one participant wrote:

*“The 5th year student and the FY1* [foundation doctor Year 1] *were very sympathetic and gave me time to discuss the event” (student no. 7).*

We found that the student no. 7’s comments about satisfactory support were not limited to fostered emotional support. Their discussion with ward staff was also associated with academic lessons that they could take forward in their professional life. The same student continued:

*“ … my colleagues were, I think, both baffled by and sympathetic to my reactions to the death, while coping with it themselves. We supported each other by discussing the event in practical ways without blaming anybody”.*

Here we can see the interplay between what we illustrated earlier – that external coping measures (i.e. speaking to someone) supported the internal coping process both in terms of personal feelings and professional roles.

Alternatively, one participant narrated a story about a patient death where they did not feel supported:

*“Everyone in medicine is so hardened by deaths and it came like nature to them, but not me who had that first experience” (student no. 6).*

Often, experiences where students did not feel supported were situations where personal emotional support was not provided. An interesting point that can be drawn here is the link between early personal emotional experience (i.e. first time) with patient death and the appreciation for support from those who were sensitive to students’ emotions. One participant’s experience took this idea further, highlighting the importance of holistic support. They wrote:

*“ … not much [support]. The conversations focused primarily on the Lessons Learned, and what should have been done better, which is an area I feel very strongly about getting right. The personal/moral/existential aspects were not really discussed” (student no. 4).*

In relation to support received from the medical school, participants also identified points of satisfactory and unsatisfactory support. Participants often talked about the support they received in preparing for their work on the wards. For instance, one participant wrote:

*“They did their best in preparing me for managing the EOL [end of life] issues. But that's the farthest they could go, I think; the rest of the lessons we have to learn from hands on experience” (student no. 2).*

Although the student no. 2 appreciated the support from medical school, their view towards it was limited only to the preparation course they received in preclinical years. Related to this point, participants also referred to the idea that they felt that medical school support was not relevant in these specific instances of experiencing patient death. One participant wrote:

*“ … at the moment of losing a patient, I thought the school was [too far away] to reach for support. They might be supportive, but I'd rather talk to the people I am working with” (student no. 8).*

### Suggestions for support

Participants talked about two key suggestions for support. The first was around timing. Students discussed the importance of support in the preparation for patient death experiences, including both practical and emotional aspects. For example, one participant criticized the skills preparation:

*“I don't think death and the accompanying tasks that need to be performed by medical staff are well covered/taught by the medical school. I think we should have a clinical skills session at some point taking us through confirming and certifying death - the practical and emotional aspects” (student no. 10).*

Second, the characteristics of good support were highlighted; these are summarized in Table 4. These themes concerned structure, activity, sensitivity, and embracing support. In other words, the support following patient death should be structured, formal and organized; it should be more active, as some students might not seek support themselves; it should be sensitive, since over a period of time experiencing a number of patient deaths, a healthcare professional may become hardened and sensitivity to medical students’ early experiences might be lost; and support from peers and near-peers can be helpful and should be embraced.

**Table 4:** Characteristics of good support

Characteristics	Examples	Quotations
Be structured	• A periodic group meeting (can be non-compulsory) • Clinical skill sessions	“A monthly group meeting between students and peers, seniors like year 5 and FYs [foundation doctors]. This could help them address this experience and have them talk it out among others” (student no. 6).
Be active	• Approach the student to check if they are ok	“Some medical students may not ask for support even though they are feeling like they need it” (student no. 6).
Be sensitive	• Be welcoming and understanding, especially for those who are having early experiences with patient death	“Be sensitive to medical students’ emotions. Be there to listen when they want to talk or share a story about the person that [has] gone. [Keeping] them in the present [is] a subtle reminder that we have to live the life we have now” (student no. 5).
Embrace peer and near-peer support	• A discussion about patient death between 4th, 5th year medical students and foundation doctors	“More peer support from [medical] students and FYs. I think a 4th year medical student witnessing patient death may want to talk to someone, but would not be comfortable speaking to a senior staff member” (student no. 4)

### Further findings from narrative analysis

We highlight the exemplar narrative written by the student no. 10 (See Table 5) for analysis, as it not only illustrates the types of experiences that students reported, but also provides insight into the interplay between reflection and learning through these experiences.

**Table 5:** Exemplar narrative

Line	Response
1 2 3 4 5 6 7 8 9 10 11 12 13 14 15 16 17 18 19	“In my last week of 5th year one of the patients we had been looking after died suddenly on the ward. The cardiac arrest alert went out but was then cancelled as he had a DNACPR [do not attempt cardiopulmonary resuscitation] in place. Having never confirmed a death before I took the opportunity to go along with the FY1 [foundation year 1] to perform this. On entering the room with the registrar and F1 [foundation year 1] there was a nurse there. The registrar began to go over the process of confirmation then left the F1 and myself to it. At this point the nurse asked if she should leave as the patient was a relative. The F1 and myself, while having said nothing inappropriate, were mortified by the whole situation and felt terrible! We moved on to confirm the death, then went to speak to the [family member] who could not have been nicer about the whole situation. I was the one to confirm the death, and while the upset with the relative happened, I feel this was overall a positive experience. It has taught me to always check identities for one thing. I feel it is very beneficial to carry out this process as a medical student, and I feel better prepared for FY1 as a consequence. I talked through this with both the FY and my supervisor, and reflected on it in my portfolio. I think it is something that should be covered more thoroughly in the curriculum - my experience was very beneficial, but was purely by chance and by actively seeking out this experience.” ***If you could change anything about the support, what would it be?*** “I don’t think death and the accompanying tasks that need to be performed by medical staff are well covered/taught by the medical school. I think we should have a clinical skills session at some point taking us through confirming and certifying death - the practical and emotional aspects.”

Bold type indicates item in questionnaire

The narrator begins their reflection on the patient death incident with an orientation of time, place and person (*last week of 5th year, on the ward, a patient who had suddenly died*, line1–2). The following five sentences concern the death confirmation process without realizing that the nurse in the room was a patient’s relative. These sentences arguably represent Labov’s [31] evaluation phase as the participant expressed their evaluation of the incident through using emotion words (i.e. *mortified and terrible*, line 8).

The narrative proceeds to the complicating action phase [31] in which the narrator performed death certification with a foundation doctor. A repetition, which is a kind of subtle emotional device [22], is found here when they repeated mentioning about the presence of patient’s relative and how they saw the situation (*upset*, line 10). Through the lens of reflection, we interpreted that these negative feelings were the first stage of the student’s reflection in which they identified discomfort in the situation.

However, when the narrative moves to the resolution and coda parts, indicating the ending of the story [30], the emotion words shift from negative to positive ones. This transition point aligns with the point within the story where the student reflects on what they have learned; they discuss what the situation has ‘*taught*’ them (line 11). From the narrator’s current point of view (explicitly signaled by the repetition of ‘*I feel*’, lines 11 and 12), this situation is good in terms of the preparation for the foundation doctor level. Adjectives and adverbs such as *positive* (line 11), *beneficial* (lines 12 and 16) and *better* (line 12) were used to indicate their feeling. The intensifier *very* was used repeatedly before beneficial (lines 12 and 15), demonstrating how they positively think about the situation overall, arguably because of what they have learned.

Reflecting on what happened, we also see how the participant felt about the practical and emotional preparation for certifying death in clinical year practice. The narrator employs the use of negation by explicitly saying, ‘*I don’t think death and the accompanying tasks that need to be performed by medical staff are well covered/taught by the medical school*’ (lines 17–18). A counterfactual thinking in the form of negation can be identified here, reflecting disappointment at an event that they think should have happened but did not [32]. Within the framework of reflection stages, we interpret that there are two new perspectives emerging from their analysis of the event: the lessons they have learned from the death certification process, and the opportunity for the medical school to improve their preparation course regarding this aspect.

## Discussion

By exploring narratives of medical students about their experiences with patient death, this study identified many issues related to the research questions.

### Coping strategies for patient death

Through thematic analysis, we observed that many coping strategies that students employed resembled the results of previous studies. Each of the strategies will now be discussed to show how the findings reflect or extend the results of existing articles.

#### Turning to religion

Turning to religion was identified as a common coping strategy for medical students [3, 5, 7], physicians [33] and nurses [34] after a patient had died. Although there are not many articles clearly explaining how religion helps with medical student coping mechanisms following patient death, Rudisill et al. [35] found a relationship between medical students’ strong religious beliefs about life after death and lower death anxiety. This could be due to the interpretation of death based on the students’ religious background; for example, some students might feel that the dying patient was being protected by a higher power, and then interpret the death in a positive way [36].

#### Staying busy

Coping by staying busy with other responsibilities might be seen to agree with the results of previous studies [3, 7, 19], showing that keeping on with clinical tasks was a way to cope with patient death. However, there might be a minor difference in this current study. Pessagno et al. [3] found that carrying on with physicians’ tasks was linked to the identification of professional attributes; that is, the students in their study kept themselves busy because they saw that “continuing to help others is an essential part of medicine regardless of how many patient deaths physicians and medical students experience” ([3], p. 54). Some students in our study described staying busy as a coping strategy, without the professional attributes necessarily being identified. On the one hand, staying busy was a way to shift awareness away from sadness they were experiencing. On the other hand, staying busy could be an action following the development of professional attributes.

#### Enduring negative emotions

This study has revealed that some students processed their negative emotions internally. This method of coping has not been mentioned much in previous studies except that of Firth-Cozens et al. [19]. In their study, 2.3% of medical students chose to let the feeling fade away through time without necessarily seeking support [19]. They also found that this coping strategy was associated with a high Fear of Death (FOD) score, which is a psychometric tool measuring the level of death anxiety [19]. Interestingly, a participant in the current study who used this strategy reported that they experienced dreams about the deceased patient that kept returning for some time. These recurrent dreams about patients who died may be interpreted as intrusive thoughts or memories that can occur to healthcare providers who are powerfully affected by the death [37, 38]. To our knowledge, our study is the first to report intrusive memories in medical students after patient death. From our findings, we also propose that there might be some connections between these features (enduring negative emotions, death anxiety and intrusive thoughts), and that these connections leave an area for further research.

### Personal re-interpretation of the experience into a lesson learned for personal life and professional role

Seeing the death in a positive light by considering life value has not generally been mentioned in the existing literature studying medical students’ coping strategies. Kelly et al. [2] briefly mentioned that the death of a young patient led to students contemplating their life values. Our study supports their finding by demonstrating how life values were contemplated, in terms of living life to the full and being thankful to people for their support.

Another personal re-interpretation of patient death experience was found in terms of professional role. Here, students have shown how experiences were turned into lessons learned for their professional role in future approaches to dying patients and patient death. This feature was found in the student no. 4’s comment in the thematic analysis (lessons about CPR cart and the presence of other patients around the death of a patient) and the student no. 10’s comment in narrative analysis (lessons about death certification process and its preparation). This shows that students themselves have the potential to interpret the EOL practice in a positive and meaningful way, aligning with the findings of MacLeod et al. [39].

It can be simply proposed that the lessons students have learned through the re-interpretation of experiences display the final stage of reflection we discussed earlier, which is the development of new perspectives. However, we found an interesting aspect from the comments of the student no. 4 (see section **Coping and learning from experiences with patient death**) and the student no. 10 (see Table 5). In both comments, the affective elements shifted from negative to positive at the end, resulting in valuable lessons for their professional role. In particular, through narrative analysis of the student no 10’s reflections, we can see how learning provides a bridge between the negative and positive emotions that medical students experience. This quality of transforming a negative emotion into a productive consequence is not only learning but, we argue, emotional agility [40]. David et al. [40] propose that emotional agility is the quality by which people approach negative emotions in a mindful, productive and value-driven way. Through reflection there is an opportunity for individuals not only to learn, but to cope through learning. More importantly, there is also an opportunity to learn to cope through developing skills such as emotional agility and mindfulness.

### Normalization

This study showed that strong feelings towards the death of a patient could be recognized as normal responses, resulting in coping mechanisms. To our knowledge, none of the existing articles have reported normalization as a coping strategy after medical students’ experiences with patient death. However, some studies suggested that nurses [41] and ambulance service workers [37] broadly employed this strategy. In medical sociology, normalization is the process in which a new social member (such as a medical student) learns the ways of thinking and internalizes the professional values and behaviors through participating in a community of practice [42]. Although this definition implies normalization as an internal process, our data suggest that interaction with other people influences normalization (see Fig. 1). Rhodes-Kropf et al. [7] also advocated that senior staff members could enhance this process by routinely asking medical students about their feelings following the death of a patient and helping them to realize that clinical practices involve strong emotions.

### Speaking to someone and re-interpretation of the experience into a lesson learned for professional role

Speaking to someone was shown to be a common method of coping after patient death, aligning with the findings of Rhodes-Kropf et al. [7] and Jones et al. [8]. Our results also correspond with existing research in that speaking to someone about patient death provided not only emotional comfort and academic benefit (2, 5, 8], but also the development of professional attributes [5]. However, this study extends existing literature as it illustrates the interplay between personal and group reflection supporting learning about professional roles. Team interaction, especially between students and experienced professionals, was shown to help the students recognize that there were both personal and professional aspects to learn and balance in relation to patient death experiences.

Another feature of this investigation that aligns with previous studies is that participants most often spoke to peers and near-peers about the death. This could be due to the fact that peer support occurs in an informal environment in which people of similar context and intellectual status are interacting [43]. However, other literature has found that peers, near-peers or senior peers are often considered trusted sources of practical advice and guidance for medical students [44].

### Suggestions for support

Our study has identified timing as being a key characteristic of good support. It was clear that students felt support was needed both before and after an experience with patient death. Beforehand, students felt that medical schools ought to prepare them for these experiences. They highlighted that the preparation process should include both the practical (e.g. death confirmation and certification) and emotional aspects around death. This aligns with work from Jones et al. [8], who also identified the importance of addressing both practical and emotional aspects of patient death.

After experiencing a patient death, students valued informal opportunities to debrief with more experienced colleagues, which aligns with other literature [8]. Importantly, our study highlighted the importance of the opportunity for students to reflect on not only the professional but also the personal and emotional aspects of patient death experiences. We also identified that the students needed structured or formalized support following the death of a patient. This reflects work by Kelly et al. [2], which suggests that formalized discussions after patient death could enhance opportunities for students to talk and share their experiences. Other literature has suggested that regular group sessions where trained faculty is included may be helpful [45] and can also be done in the form of non-written reflection e.g. Schwartz rounds [46].

In addition to considering the timing of support, being empathetic to the student experience was identified as important. Empathy, defined by Rogers in 1961 [cited in [47]], is the capacity to be sensitive to what others are feeling and to be able to communicate sensitively with them. As discussed in other work, the reaction of the medical staff, who may have seen patient death many times, could be very different from that of inexperienced medical students [9]. As reflected in both our study and Rhodes-Kropf et al.’s [7] findings (see Table 2), students sometimes felt the healthcare team had become so hardened by death that they lacked the sensitivity to acknowledge it with students. As mentioned above, normalization is an important part of coping for these medical students, and staff being empathetic to students’ experiences may be helpful in this process. Some of these findings might also explain why students highlight speaking to peers and near-peers as being particularly valuable. Near-peers may be able to understand and empathize better with the challenges and difficulties medical students are facing; therefore, they are more able to support and give appropriate suggestions [48, 49].

The last characteristic of good support is for medical educators to take an active role. A student might not initiate a conversation, even though he or she needs to be supported. This finding aligns with the results of Pessagno et al. [3] and Smith-Hans et al. [9], showing that students appreciated the support actively initiated by faculty members. In general, regular active support from faculty has been shown to be beneficial, because many students are unlikely to seek help from faculty even if they are having difficulties [50].

### Strengths and key limitations of this research

This study has several strengths. First, it was carefully designed to have an alignment between epistemology, methodology and method to show internal coherence, which is an important quality of rigorous qualitative research [51]. Second, we employed group approaches in the data analysis process. The reliability of data analysis [52] and interpretative rigor were therefore enhanced by researcher triangulation [53]. Third, multiple analytical methods, including thematic framework analysis and narrative analysis, were used. This denotes the adoption of data analysis triangulation, which helps promote a deeper and holistic understanding of an interested phenomenon and enhance the validity of qualitative research [54]. Finally, through an interpretivist research paradigm, there is a recognition that there are multiple interpretations of our social world and no single way of knowing [21]. Therefore, the researcher’s background and position could have impacted the research and a number of approaches were taken to enhance reflexivity to address this. In addition, every comment from the supervisor and research team was kept in the main researcher’s reflexive journal throughout the study process.

Nevertheless, our study contains limitations. Twelve participants is a small sample size and our method of sampling could have missed some key people, such as extreme cases or outliers [55]. However, being qualitative research, this study aimed to explore deeply the medical students’ experiences around their most memorable patient death. Malterud et al. [56] suggest that qualitative researchers should consider information power in relation to sample size. We would argue that the factors which contribute to information power are favorable. Not only were our data able to address our research questions, which were narrow in focus, but the content was rich. Responses from our specific group of participants were narratives about experiences with patient death, averaging 400 words, demonstrating that the responses we received were not just short comments. Although word counts do not directly reflect data richness, there is an association between longer word counts and other dimensions of data richness (e.g. personal responses and responses with specific knowledge [57, 58]). It can be argued that our narratives were filled with personal reflections on patient death and with specific medical knowledge included (such as the readiness of the cardiopulmonary resuscitation cart and the death certification process). Therefore, the richness of data that answers the research questions and our rigorous analytical methods are a prime requirement, rather than statistical representativeness or meeting the controversial criteria of saturation [56, 59].

Being a study conducted at a single institution, caution must be taken in making widespread generalizations. Qualitative research in itself is not concerned with generalizability [60], but transferability is important. Transferability is the extent to which research findings are relevant, useful or able to inform other similar settings or contexts [53]. To enhance transferability, comparisons between our results and those of the existing literature were thoroughly considered [61], and we found many relevant points that added to existing knowledge. Also, the study’s details, such as settings, theoretical perspective, methodology, sampling and analyses, were provided in order for readers to judge themselves whether this study can be applied to their contexts [61]. We hope that the potential transferability of this study could contribute to a wider range of healthcare education practices relevant to multiple learner groups (e.g. medical students, nursing students) especially in the area of student support regarding patient deaths.

Another limitation is that we did not explicitly ask participants about past experiences with death and dying prior to the clinical year training. Existing literature suggests that previous close personal bereavement, like the death of a close friend or a family member, could both positively or negatively affect how medical students approach patient death [62], depending on how well the bereavement is processed. Furthermore, cadaveric dissection in preclinical years can affect how students cope and learn from their feelings around death [3, 7]. Although our questionnaire was open ended in nature, none of our participants connected their clinical patient death experiences with personal experiences such as a death in the family or with previous experiences in anatomy dissection. Future research could explore this area more explicitly.

### Recommendations for future research

As well as anatomy dissection, there are three key areas worth highlighting for further research. First, some participants endured emotional pain after patient death and reported intrusive memories that came in the form of recurrent dreams. Firth-Cozens et al. [19] found an association between enduring negative emotions and death anxiety, and we found a potential link between enduring negative emotions and intrusive memories. Further exploring this relationship may be an area for future research. Second, we identified links to mindfulness and emotional agility with the coping experience of medical students. Many articles refer to the use of mindfulness in medical education in terms of stress and distress management [63, 64]. However, none of them specifically studied how mindfulness and practices for coping with patient death could be taught as a part of emotional agility.

Third, our study has not resolved the extent to which ward staff and the medical school are ready to be supportive after patient death. We have studied medical student perspectives in this regard and found that they appreciated support from peers and near-peers, including foundation doctors. However, data from the perspectives of others concerning their supportive roles have not yet been collected. Data from them could add richness to – and address gaps in – the existing literature, such as that called for by Wear [65].

## Conclusion

Our research provides insights into medical students’ most memorable experiences with patient death in clinical placements. This included the first death, unexpected death and expected death of a patient with whom the student had a connection. We found that students employed many internal and external coping strategies. Within those coping strategies, student reflections were shown to be important for the development of lessons learned from patient death. Through their reflections, some students were able to balance these two sides and learned to act upon the incident maturely. Others showed they could come up with valuable lessons on their own, despite having negative feelings at the beginning, demonstrating emotional agility. Importantly, our results also showed that support from ward staff often enhanced reflections by helping students recognize both personal/emotional and professional aspects of the experience. Yet, participants also gave voice to how they could be better supported as reflective learners in a reflective learning environment.

We hope that these finding will highlight the importance of reflection for medical students to learn and grow from experience. We suggest that undergraduate medical teachers consider promoting a reflective and supportive environment around patient death experiences with the ultimate aim of equipping medical students with helpful coping strategies as they develop as professionals in their own right.

## Supplementary information

**Additional file 1.** Online questionnaire. Question items in questionnaire.

## Data Availability

As data sharing was not stipulated in the approved ethics committee application, data for this paper will not be shared. We did not have consent from the participants to do so.

## Abbreviations

BOS: Bristol Online Survey
CPR: Cardiopulmonary resuscitation
EOL: End of life
FY: Foundation year doctor
