# Supplementary material for: Learning and coping through reflection: exploring patient death experiences of medical students
Source: BMC Med Educ. 2019 Dec 4;19:451. doi: 10.1186/s12909-019-1871-9 (PMC6894273; doi:10.1186/s12909-019-1871-9)
Supplement: Supplementary file 1 — Additional file 1. Online questionnaire. Question items in questionnaire. [file 12909_2019_1871_MOESM1_ESM.pdf]

Online Questionnaire

1. In the context of your clinical placements in year 4 and/or year 5, we would like you to think about your experiences related to patient death. When we say ‘experiences related to patient death’, this could be a direct or indirect experience. For instance, this could include a situation where you have worked with a patient and later found out they have passed away.

   Please choose your **most memorable experience** related to patient death and describe it below. Consider these prompts in your description:

   - What happened?
   - When and where did it happen?
   - How did you feel about the experience?
   - How did you cope with it?
   - What did you learn from the experience?
   - How do you feel about the experience now in hindsight?

2. Did you speak with anyone about the experience?

   ○ Yes   ○ No (If you select "No", then go to question 3)

   2.1. Who did you speak to?

   2.2. Was this someone you approached or did they approach you?

   ○ I approached them   ○ They approached me

   2.3. How did this conversation influence your perspective on the experience?

3. Related to the experience you have described, how well supported did you feel by the followings?

   3.1. Ward staff (people you worked with on the ward: attendings, seniors, registrars, colleagues, nurses and other medical staff)

   ○ Very supported   ○ Somewhat supported   ○ Slightly supported

   ○ Not at all supported   ○ Not applicable

   Please explain why you felt that way:

3.2. Medical school (people from the medical school who were not involved in the ward setting where the experience took place: mentors, preceptors, educational supervisors and clinical supervisors. This also includes support systems provided by the medical school)

○ Very supported ○ Somewhat supported ○ Slightly supported

○ Not at all supported ○ Not applicable

Please explain why you felt that way:

4. If you could change anything about the support, what would it be?

4.1. In this instance

4.2. When a medical student is experiencing patient death in their clinical placements more generally.

5. Your gender
   - ○ Male
   - ○ Female
6. Age
   - ○ 18-19
   - ○ 20-24
   - ○ 25-29
   - ○ 30-34
   - ○ 35-39
   - ○ 40+
7. What year in medical school are you?
   - ○ Year 4
   - ○ Year 5
8. Is English your first language?
   - ○ Yes
   - ○ No
